# Clustered Regularly Interspaced Short Palindromic Repeat-1 (CRISPR-1) Locus as a Tool for Tracing the Zoonotic History of Salmonella enterica Strains

**DOI:** 10.7759/cureus.62050

**Published:** 2024-06-10

**Authors:** Maan Neamah, Evan Mahdi, Muhammed Sameir, Safin Hussein, Abdulmalik Saber

**Affiliations:** 1 Department of Medical Biotechnology, Al-Qasim Green University, Babil, IRQ; 2 Department of Medical Laboratory Techniques, Altoosi University College, Najaf, IRQ; 3 Hammurabi College of Medicine, University of Babylon, Babil, IRQ; 4 Department of Biology, University of Raparin, Sulaymaniyah, IRQ; 5 Department of Psychiatric and Mental Health Nursing, Hawler Medical University, Erbil, IRQ

**Keywords:** genotyping, sequencing, zoonotic transmission, intergenic spacers, crispr, salmonella enterica strains

## Abstract

Background

*Salmonella enterica* is a significant foodborne pathogen that causes considerable illness and death in humans and animals. The clustered regularly interspaced short palindromic repeat (CRISPR)-CRISPR-associated protein (Cas) system in bacteria acts as an adaptive immune defense against invasive genetic elements by incorporating short intergenic spacers (IGSs) into CRISPR loci. These loci serve as molecular records of past interactions with phages and plasmids, providing insights into the transmission and evolution of bacterial strains across different hosts.

Aim

This study aimed to investigate the diversity of IGSs in the CRISPR-1 locus of *S. enterica* isolates from humans and camels. The objective was to assess the potential of IGSs to distinguish strains, track sources, and understand patterns of zoonotic transmission.

Materials and methods

Genomic DNA was extracted from multiple strains of *S. enterica*, and the CRISPR-1 locus was polymerase chain reaction (PCR) amplified and sequenced. The sequences were compared to identify distinct patterns of IGSs and potential host-specific characteristics. Sanger sequencing and bioinformatics tools were used to classify the IGSs and determine their similarity to known sequences in the National Center for Biotechnology Information (NCBI) database.

Results

Sequence analysis revealed five distinct CRISPR-1 types among *S. enterica* isolates from humans and three among camel isolates. The presence of shared IGSs between human and camel *S. enterica *isolates suggested zoonotic or reverse-zoonotic transmission events. Additionally, host-specific unknown IGSs (UIGS) were identified. Importantly, camel isolates initially identified as *S. enterica* subspecies *enterica* serovar Enteritidis based on rrnH gene sequencing were reclassified as *S. enterica* serovar Enteritidis based on CRISPR-1 profiling, demonstrating the higher resolution of CRISPR-based genotyping.

Conclusion

The diversity of IGSs in the CRISPR-1 locus effectively differentiated *S. enterica* strains and provided insights into their evolutionary origins and transmission dynamics. CRISPR-based genotyping proves to be a promising tool to complement traditional serotyping methods, enhancing the molecular epidemiology of salmonellosis and potentially leading to better management and control strategies for this pathogen.

## Introduction

Serovars of *Salmonella enterica* are significant pathogens in the global foodborne context, causing a considerable number of cases of gastroenteritis and resulting in thousands of deaths annually [1]. This bacterium has developed various defense mechanisms against bacteriophages, which can infect and lyse bacterial cells. As a result, bacteria have evolved an array of mechanisms to resist viruses. Among the most complex defense systems is the clustered regularly interspaced short palindromic repeat (CRISPR)-CRISPR-associated protein (Cas) system, which functions as an adaptive immune system, providing sequence-specific protection against invasive genetic elements such as phages and plasmids [2,3]. This defense mechanism is intricately connected to the pathogen's capacity to survive and proliferate in diverse hosts, underscoring the importance of comprehending its transmission dynamics. 

The mechanisms and epidemiology of zoonotic and reverse-zoonotic transmission of *S. enterica* are crucial for understanding how this pathogen spreads among different hosts and environments. Zoonotic transmission, which occurs when pathogens are transferred from animals to humans, has been extensively studied for *S. enterica*. This transmission usually occurs when people ingest contaminated food, come into direct contact with infected animals, or are exposed to animal waste in the environment [4]. Serovars like *Salmonella *Enteritidis and *Salmonella *Typhimurium are commonly found in poultry and livestock and can cause outbreaks of foodborne illnesses in humans [5]. Additionally, these bacteria can carry antibiotic resistance genes, making treatment and control efforts more challenging. Recent studies emphasize the need for comprehensive surveillance and the implementation of biosecurity measures in animal farming to reduce the risk of zoonotic transmission to humans [6,7].

Reverse-zoonotic transmission, also known as anthroponosis, involves the transfer of pathogens from humans to animals. While not as well-documented as zoonotic transmission, this occurrence poses significant threats to animal well-being and has the potential to impact biodiversity. There have been reports of reverse-zoonotic events involving *S. enterica*, where strains originating from humans have been transmitted to animals, establishing new reservoirs of infection. Notably, instances of *S. enterica* isolates sourced from humans have been detected in animals, suggesting a complex interplay and bidirectional exchange of pathogens [8,9]. In these transmission pathways, the CRISPR-1 locus within *S. enterica* plays a vital role as a genetic repository of past interactions with phages and plasmids. Consequently, it facilitates the tracking of the pathogen's transmission history and evolutionary trajectory.

Understanding both zoonotic and reverse-zoonotic transmission mechanisms is crucial for developing effective control strategies. Enhanced genoserotyping techniques, such as CRISPR-based genotyping, offer higher resolution when tracking transmission routes compared to traditional methods. These techniques can identify specific intergenic spacers (IGSs) that act as molecular markers for different host interactions, providing valuable insights into the spread and persistence of the pathogen across human and animal populations. This knowledge is essential for formulating policies and implementing interventions aimed at controlling the spread of *Salmonella* and safeguarding public health. At the core of these genoserotyping techniques lies the CRISPR-Cas system.

The CRISPR-Cas system operates by integrating short IGS sequences into the bacterial genome's CRISPR loci. These IGSs come from exogenous genetic elements and are inserted at regular intervals among repetitive sequences, acting as molecular records. This mechanism of information storage allows the bacterial cell to identify and degrade future encounters with the same or similar genetic material [10]. The CRISPR loci themselves serve as a unique repository of past interactions between bacteria and invasive genetic components. This valuable information helps us understand bacterial population dynamics and their adaptive responses to environmental conditions over time [11].

Understanding the diversity and evolution of CRISPR loci in *S. enterica* is important for several reasons. Firstly, it can advance genotyping and source-tracking methods, which are crucial for epidemiological investigations and identifying potential contamination sources [12]. Additionally, it can provide valuable insights into the transmission of *Salmonella* strains among humans, animals, and other reservoirs, improving our understanding of pathogen transmission dynamics.

CRISPR loci are an ideal tool for studying zoonotic or reverse-zoonotic transmission because bacteria can acquire genetic material from viruses or other mobile genetic elements found in various host environments. This enables them to effectively capture their previous interactions. For instance, *Salmonella* strains can acquire and incorporate intergenic sequences from phages or plasmids that are specific to human, animal, and environmental reservoirs [13]. As a result, their CRISPR loci retain a genetic signature that can reveal their transmission patterns and evolutionary origins.

The primary goal of this study was to discover new arrays within *Salmonella* CRISPR loci and evaluate their efficacy in differentiating strains and tracing their sources. We aimed to develop a distinctive genoserotyping system using CRISPR profiles that could potentially enhance or outperform current methods. Furthermore, we examined the zoonotic relationship between *Salmonella* strains in humans and camels by comparing their CRISPR IGS content.

## Materials and methods

Isolates of *Salmonella*

The Department of Veterinary Microbiology, Faculty of Veterinary Medicine, University of Kufa in Iraq has provided 20 bacterial isolates for this study. These isolates include two subspecies of *S. enterica*: 10 isolates of *S. enterica* subsp. *enterica *serovar Enteritidis obtained from humans and 10 isolates of *S. enterica* subsp. *enterica *serovar Indiana obtained from camels. The isolates were incubated overnight at 37°C in LB medium.

Genomic DNA extraction

The sediments from fresh *Salmonella *isolate cultures in LB broth media were obtained by centrifugation at 14,000 rpm for two minutes. The bacterial genomic DNA was extracted using a commercial DNA extraction kit (G-spin Genomic DNA Extraction Kit for Bacteria, 17121, Intron, Korea) following the manufacturer's instructions. The quality and quantity of the extracted DNA were assessed using a Quantus™ fluorometer (Promega, USA).

CRISPR-1 locus amplification and sequencing

The PCR was able to copy the CRISPR-1 locus region with a forward primer (CRSPR-1F) (5′-CGGAGTCGGAAACGTAGTAATG-3′) and a reverse primer (CRSPR-1R 5′-GAATGAGTGACGCTGAGAAGAA-3′). Primers were designed using Primer3. The PCR reactions were carried out using an Optimus 96G thermal cycler (QLS, UK) under the following conditions: initial denaturation at 94°C for five minutes, followed by 35 cycles of denaturation at 94°C for 30 seconds, annealing at 56°C for 30 seconds, extension at 72°C for four minutes, and a final extension step at 72°C for 10 minutes. Agarose gel electrophoresis was used to separate the amplified PCR products. The gel used was 1.5%, and the DNA dye used was RedSafe (Intron, Korea). The electrophoresis conditions were set at V: 90 for a duration of 42 minutes. A DNA ladder was included as a marker. Subsequently, the amplified PCR bands were concentrated using the Wizard® SV Gel and PCR Clean-Up System (A9281, Promega, USA), followed by sequencing.

**Figure 1:**
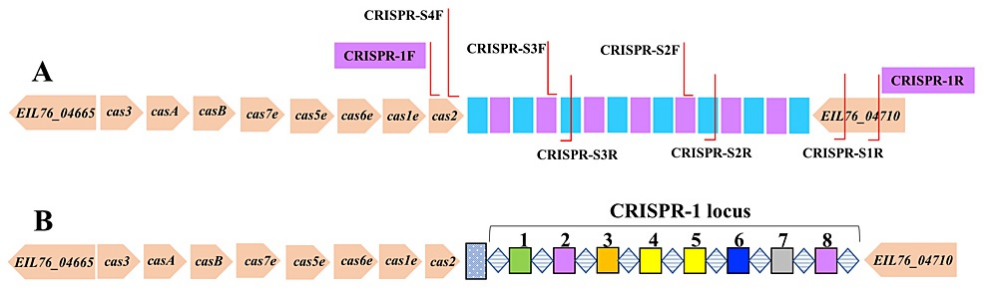
Structural gene map of the CRISPR-1 locus. The CRISPR locus is flanked by the EIL76_04665 and EIL76_04710 genes. To differentiate between amplification primers and sequencing primers, two lavender boxes indicate the positions of the amplification primers (A). The DRs of the CRISPR-1 locus in *Salmonella* strains are depicted by diamond boxes with light-blue horizontal lines, while the IGSs are represented by colored squares (B). CRISPR: clustered regularly interspaced short palindromic repeats; DRs: direct repeats; IGSs: intergenic spacers

Sanger DNA sequencing (Macrogen, Korea) was performed using the following sets of primers: CRSPR-S1R (5′-CGGAGTCGGAAACGTAGTAATG-3′), CRSPR-S2F (5′-GCCACCCTCGGCTTTAAT-3′), CRSPR-S2R (5′-GAGTAACGTGCGCTGGAA-3′), CRSPR-S3F (5′-CTGTTGCATTAGATTCGTGTTCC-3′), CRSPR-S3R (5′-CCTGTACGCCTCAGGTTTATC-3′), and CRSPR-S4F (5′-CGTATTCCGGTAGATBTDGATGG-3′). The gene map and primer positions are shown in Figure 1A. The semi-conserved CRISPR-1 locus in *S. enterica* subsp. *enterica *serovar Typhimurium ATCC14028 (accession number: CP034230) has been extensively studied and serves as a prominent model due to its clearly defined gene map (Figure 1B).

## Results

Organization and structure of the CRISPR-1 locus

Out of a total of 20 samples of *S. enterica*, the CRISPR-1 region of 17 (90%) was successfully amplified by PCR. Of these, 10 isolates were from humans, and seven samples were from camels. The PCR amplification showed distinct sizes of 700-800 bp PCR bands for the CRISPR-1 region in the positive samples. The CRISPR-1 region can be divided into two sub-regions: direct repeats (DRs) and IGSs of variable sequences (Figure 1B) [14]. The results revealed a wide range of CRISPR-1 region content among the isolates, regardless of whether they were from similar or different hosts. However, because the number of DRs and IGSs in the CRISPR-1 locus varies among or within species, this structure remains semi-conserved across all *S. enterica* strains.

Analysis of CRISPR-1 sequences

**Figure 2:**
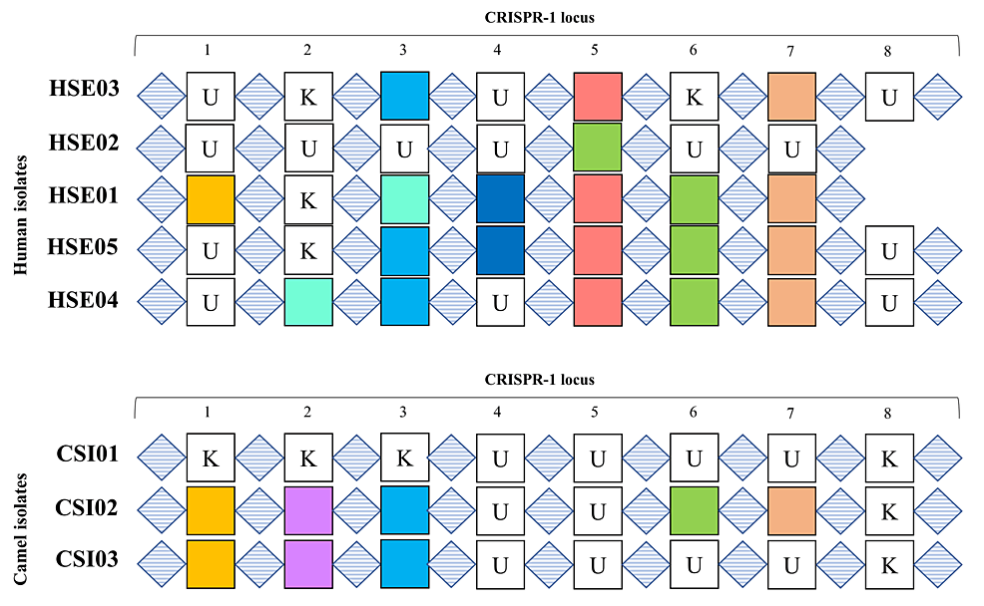
The alignment of CRISPR-1 loci across all sets of Salmonella isolates. Diamond boxes with light-blue horizontal lines depict the DRs, while colored squares represent the IGSs. Shared IGSs are indicated by squares of the same color, whereas white squares represent unshared IGSs among different sets. Any white square labeled with the letter "K" signifies that the corresponding IGS is associated with a known sequence in the NCBI database. Conversely, white squares labeled with the letter "U" indicate that the corresponding IGS does not have a match to any known sequence in the NCBI database. CRISPR: clustered regularly interspaced short palindromic repeats; HSE: human *Salmonella* Enteritidis; CSI: camel *Salmonella* Indiana; NCBI: National Center for Biotechnology Information; DRs: direct repeats; IGSs: intergenic spacers

The Sanger sequencing method was used to determine the sequence of the CRISPR-1 locus in a total of 17 positive samples. The findings revealed that the sequences of the human-isolated *S. enterica *can be classified into five primary types (HSE01, HSE02, HSE03, HSE04, and HSE05), while the camel-isolated strains can be grouped into three main sequences (CSI01, CSI02, and CSI03). The arrangement and structure of the CRISPR-1 sequence in *Salmonella* are derived from a human isolate. The sequences of the CRISPR-1 region in human-isolated strains displayed two distinct patterns. Pattern-HA showed eight DRs and seven IGSs (HSE01 and HSE02), whereas Pattern-HB consisted of nine DRs and eight IGSs (HSE03, HSE04, and HSE05) (Figure 2).

The IGSs of all isolates were examined to identify potential similarities and matches with previously sequenced *Salmonella* genomes in the National Center for Biotechnology Information (NCBI) database. Consequently, seven IGSs were discovered in HSE01 of Pattern-HA that were exactly the same as the CRISPR-1 IGS sequences already known. In contrast, HSE02 only had one IGS (IGS5) that matched a known *Salmonella*-CRISPR-1 sequence. Several single nucleotide polymorphisms (SNPs) were observed in the remaining IGSs at that locus. These SNPs do not appear to correspond to any known IGSs in the NCBI database. As a result, these spacers were labeled as unknown IGSs (UIGSs). The number of known IGSs and UIGSs is summarized in Table 1. Similar findings were also reported in isolates of Pattern-HB.

**Table 1:** Number of CRISPR-1 IGSs and UIGSs in all sets of Salmonella isolates. CRISPR: clustered regularly interspaced short palindromic repeats; IGS: intergenic spacer; UIGS: unknown intergenic spacer; HSE: human *Salmonella *Enteritidis; CSI: camel *Salmonella *Indiana

Set	Total number of IGSs	Number of known IGSs	Number of UIGSs
HSE01	7	0	7
HSE02	7	1	6
HSE03	8	5	3
HSE04	8	5	3
HSE05	8	6	2
CSI03	8	4	4
CSI03	8	6	2
CSI03	8	4	4

Following the analyses conducted on *Salmonella *isolates from humans, we also examined the CRISPR-1 region of *Salmonella* obtained from camels. This examination involved assessing the abundance of IGSs and comparing them to the established *Salmonella* CRISPR-1 sequences documented in the NCBI database. Although different from isolates sourced from humans, the three sets of CRISPR-1 loci in camel-isolated strains consistently consisted of nine DRs and eight IGSs. However, there was variability in the similarity of the three sets to the established *Salmonella* CRISPR-1 sequences.

Sharing IGSs is common among isolates from the same host

We analyzed IGSs and DRs in various isolates from the same host. Human-isolated strains showed a high frequency of IGS sharing. However, the arrangement or positioning of the shared IGSs appeared to be unusual. Specifically, IGS3 of the CRISPR-1 locus in set HSE01 was 97% similar to IGS2 of set HSE04. Additionally, IGS5 in set HSE02 was found to be identical to IGS6 in sets HSE01, HSE04, and HSE05 (Table 2; Figure 2). On the other hand, the CRISPR-1 locus in sets CSI02 and CSI03 of camel isolates shared the first three IGSs (IGS1, IGS2, and IGS3) (Table 3; Figure 2).

**Table 2:** Shared IGSs of the CRISPR-1 locus in Salmonella isolates from humans. The IGSs in the same row are identical, regardless of the actual IGS number. CRISPR: clustered regularly interspaced short palindromic repeats; HSE: human *Salmonella *Enteritidis; IGS: intergenic spacer

*S. enterica* isolates from humans					
HSE01	HSE02	HSE03	HSE04	HSE05	Identity
			IGS3	IGS3	100%
IGS3			IGS2		97%
IGS4				IGS4	100%
IGS5		IGS5	IGS5	IGS5	100%
IGS6	IGS5		IGS6	IGS6	100%
IGS7		IGS7	IGS7	IGS7	100%

**Table 3:** Shared IGSs of the CRISPR-1 locus in Salmonella isolates from camels. The IGSs in the same row are identical, regardless of the actual IGS number. CRISPR: clustered regularly interspaced short palindromic repeats; CSI: camel *Salmonella *Indiana; IGS: intergenic spacer

*S. enterica* isolates from camels			
CSI01	CSI02	CSI03	Identity
	IGS1	IGS1	100%
	IGS2	IGS2	100%
	IGS3	IGS3	100%

Sharing IGSs is common among isolates from different hosts

The IGSs were also analyzed to determine if there were any common IGSs among isolates from different hosts. The results showed that the CRISPR-1 locus in human isolates had specific IGSs that were also found in isolates obtained from camels (Table 4; Figure 2).

**Table 4:** Common IGSs of the CRISPR-1 locus of isolates from humans and camels. The IGSs in the same row are identical, regardless of the actual IGS number. CRISPR: clustered regularly interspaced short palindromic repeats; HSE: human *Salmonella *Enteritidis; CSI: camel* Salmonella* Indiana; IGS: intergenic spacer

*S. enterica* isolates from humans					*S. enterica* isolates from camels			Identity
HSE01	HSE02	HSE03	HSE04	HSE05	CSI01	CSI02	CSI03	
IGS1						IGS1	IGS1	100%
		IGS3	IGS3	IGS3		IGS3	IGS3	100%
IGS6	IGS5			IGS6		IGS6		100%
IGS7		IGS7	IGS7	IGS7		IGS7		100%

Isolated individuals from the same host also share unknown IGSs

Finally, we conducted an investigation to determine whether there were any UIGSs that were shared between isolates from humans and camels. However, our findings showed that UIGSs are specific to the host and are only shared among individuals of the same host species. Specifically, HSE03 and HSE05 shared UIGS1, while HSE03 and HSE04 shared UIGS2 and UIGS3 (Table 5). It is worth noting, though, that the isolates from camels exhibited a different pattern. The sets of CSI01 and CSI03 shared three distinct UIGSs but did not share any UIGSs with CSI02 (Table 6).

**Table 5:** Common UIGSs of the CRISPR-1 locus of S. enterica isolates from humans. The IGSs in the same row are identical, regardless of the actual IGS number. CRISPR: clustered regularly interspaced short palindromic repeats; UIGS: unknown intergenic spacer; HSE: human *Salmonella *Enteritidis

*S. enterica* isolates from humans				
HSE01	HSE02	HSE03	HSE04	HSE05
		UIGS1		UIGS1
		UIGS2	UIGS2	
	UIGS6	UIGS3	UIGS3	UIGS2

**Table 6:** Common UIGSs of the CRISPR-1 locus of S. enterica isolates from camels. The IGSs in the same row are identical, regardless of the actual IGS number. CRISPR: clustered regularly interspaced short palindromic repeats; UIGS: unknown intergenic spacer; CSI: camel *Salmonella *Indiana

*S. enterica* isolates from camels		
CSI01	CSI02	CSI03
UIGS1		UIGS1
UIGS2		UIGS2
UIGS3		UIGS3

Analysis of CRISPR-1 provides different identifications from the rrnH region

The analysis of the CRISPR-1 locus contents in human and camel *S. enterica* isolates yielded different results. For isolates from humans, the identified content matched the CRISPR-1 loci, confirming their classification as *S. enterica* subsp. *enterica *serovar Enteritidis. This classification was further confirmed through 16S rRNA sequencing and the conventional O and H serotyping systems. In contrast, analysis of the rrnH region sequence using the same DNA sequencing technique revealed that the isolates from camels were identified as *S. enterica* subsp. *enterica *serovar Indiana. Surprisingly, the CRISPR-1 content in camel isolates showed a closer resemblance to that of *S. enterica* subsp. *enterica *serovar Enteritidis rather than serovar Indiana (with an identity of 89% for CSI01, 93% for CSI02, and 89% for CSI03), as documented in Table 7.

**Table 7:** Analysis of the sequences of the CRISPR-1 locus of the tested isolates. CRISPR: clustered regularly interspaced short palindromic repeats; HSE: human *Salmonella* Enteritidis; CSI: camel *Salmonella* Indiana

Set	Source	Identification system	
		rrnH	CRISPR-1 locus
HSE01	Human	*S. enterica* serovar Enteritidis	*S. enterica* serovar Enteritidis
HSE02	Human	*S. enterica* serovar Enteritidis	*S. enterica* serovar Enteritidis
HSE03	Human	*S. enterica* serovar Enteritidis	*S. enterica* serovar Enteritidis
HSE04	Human	*S. enterica* serovar Enteritidis	*S. enterica* serovar Enteritidis
HSE05	Human	*S. enterica* serovar Enteritidis	*S. enterica* serovar Enteritidis
CSI01	Camel	*S. enterica* serovar Indiana	*S. enterica* serovar Enteritidis
CSI02	Camel	*S. enterica* serovar Indiana	*S. enterica* serovar Enteritidis
CSI03	Camel	*S. enterica* serovar Indiana	*S. enterica* serovar Enteritidis

## Discussion

The study has identified five distinct CRISPR-1 types among human *S. enterica* isolates and three among camel isolates. The presence of shared IGSs indicates the possibility of zoonotic or reverse-zoonotic transmission events. For several decades, the gold standard method for identifying different strains of *Salmonella* has been serotyping with specific antisera [15]. However, this method is only available in a limited number of high-quality laboratories and is applicable to a small subset of *Salmonella* strains [16]. As a result, researchers encounter challenges when trying to identify a wide variety of *Salmonella* strains that go beyond the well-known ones, such as Typhi, Paratyphi, Typhimurium, and Enteritidis [17].

There are two commonly used protocols for molecular serotyping. The first protocol examines two genes, fliC and fljB, which play a role in flagella production [18]. The second protocol focuses on genes in the rfb locus, which is responsible for the production of O-polysaccharides [19]. From a genetic standpoint, the rfb locus is approximately 8-23 kb in length, making it challenging to amplify using a conventional PCR protocol. Additionally, this system has limitations in accurately identifying the majority of *Salmonella* O serogroups and lacks sufficient discriminatory power between the identified *Salmonella* O serogroups [14].

The *Salmonella* CRISPR-1 locus IGSs provide a historical record that may indicate a potential reverse-zoonotic pathway. Although this study only examined a limited number of complete CRISPR-1 locus sequences, these sequences offered valuable and significant data for tracing the history of the tested isolates. There was a noticeable variation in the number of IGSs among the human-isolated strains, as well as shared IGSs across the five sets. It is uncommon for an isolate to possess CRISPR-1 with two copies of the same IGSs. Notably, the HSE01 set was the only sequence of the CRISPR-1 locus to contain two identical IGSs, specifically IGS1 and IGS2. We observed that HSE01 and HSE02 had a total of seven IGSs, while the other three sets had eight IGSs. This suggests that the isolates from these two sets may have encountered fewer hosts or predators compared to isolates exhibiting the eight-IGSs trait.

Phages are viruses that selectively infect bacterial hosts. Bacteria can evade phages through various mechanisms, such as CRISPR [20]. As a result, when bacteria encounter fewer hosts, they may have reduced exposure to phages and fewer IGSs. On the other hand, encountering a greater number of hosts would have the opposite effect [21]. Based on this hypothesis, it is suggested that sets HSE03, HSE04, and HSE05 have encountered a higher number of hosts compared to HSE01 and HSE02.

There are three identical IGSs shared by HSE01 from humans and CSI02 from camels, indicating a zoonotic origin. However, strains of *S. enterica* subsp. *enterica *serovars Typhimurium, Gallinarum, Dublin, Ohio, Javiana, and Pullorum share an additional IGS (IGS8) with CSI02. In fact, the majority of these strains are animal strains, particularly serovars Gallinarum and Pullorum, which are two *Salmonella* strains specific to poultry [22].

The strains in set HSE01 likely originated from humans and were subsequently transmitted to animals. This transmission occurred before acquiring the additional IGS8 through reverse zoonosis. The CRISPR-1 locus, which acts as the bacterial "memory cells," supports this idea. It has been observed that the CSI02 isolate from camels shares IGS7, which is the most common IGS among all sets originating from humans. This finding further strengthens the hypothesis that the isolates followed a reverse-zoonotic pathway. Various types of bacteria, viruses, and parasites, such as methicillin-resistant *Staphylococcus aureus*, *Cryptosporidium parvum*, and *Ascaris lumbricoides*, have been known to cause reverse zoonosis [23].

The rrnH region has limitations in identifying *Salmonella* serovars. The difference between the rrnH and CRISPR-1 loci profiling raises concerns about the reliability of commonly used molecular identification systems for *Salmonella*. Extensive research has been conducted on the effectiveness of the rrnH system in identifying *Salmonella* species and serovars. While the rrnH system can accurately identify *Salmonella* at the genus level with high sensitivity and specificity, it is important to note that the system alone cannot consistently differentiate between different *Salmonella* serovars [24].

The differences observed between CRISPR-1 profiling and rrnH identification may be attributed to the divergent evolutionary pressures that shape these genomic loci. It has been suggested that the CRISPR region evolves at a faster pace and captures strain-specific interactions with mobile genetic elements across various hosts [25]. Moreover, the CRISPR-Cas system serves as a key component of bacterial immunity against phages and other foreign DNA, thereby potentially rendering the CRISPR loci more indicative of the adaptive responses of strains to diverse environments and host reservoirs compared to the relatively conserved rrnH region.

Additionally, our study has revealed that the CRISPR-1 locus of the Indiana serovar contains a greater number of IGSs compared to the Enteritidis serovar. It is important to note that there are no common IGSs between these two serovars. This could potentially explain the discrepancy between the identity of rrnH and the contents of the CRISPR-1 locus, giving the CRISPR-1 locus an advantage over the rrnH system. The prevalence of *Salmonella *Enteritidis, in contrast to *Salmonella* Indiana strains, is higher among camels bred in the Middle East region [26,27]. Overall, it appears that the CRISPR-1 locus poses a challenge to the rrnH identification system.

This study is limited by its small sample size and potential biases in isolate selection, with a main focus on human and camel sources. This may not fully represent the genetic diversity of *Salmonella*. Future research should address these limitations by increasing the sample size and including isolates from a broader range of hosts and environments. By incorporating advanced molecular techniques, such as whole genome sequencing, and integrating molecular data with epidemiological and ecological information, a more comprehensive understanding of *Salmonella *transmission and evolution can be obtained. Ultimately, this would enhance control and prevention strategies.

## Conclusions

Our findings have revealed the presence of several short, yet variable, IGS sequences that are specific to either human isolates or camel *Salmonella* isolates. However, we have also discovered that some of these variable sequences are shared between human and camel isolates. This suggests the possibility of zoonotic or reverse-zoonotic origins. Throughout the zoonotic or reverse-zoonotic journey of the *Salmonella* strains, each host recognizes these short, variable sequences as unwanted predators, predominantly phages. This recognition leads to the immunization of the infected ancestor against these predators. We believe that this discovery will contribute to identifying the accurate origin of *Salmonella* isolates worldwide, as well as providing a reliable method for serotyping different *Salmonella* strains.
